# The myth of pulmonary metastasectomy

**DOI:** 10.1038/s41416-020-0927-2

**Published:** 2020-06-16

**Authors:** Fergus Macbeth, Lesley Fallowfield

**Affiliations:** 1 Centre for Trials Research, Cardiff University, Cardiff, CF14 4YS UK; 2 Sussex Health Outcomes Research & Education in Cancer (SHORE-C), University of Sussex, Sussex, UK

**Keywords:** Colorectal cancer, Surgical oncology

## Abstract

Pulmonary metastasectomy is widely and increasingly practiced in the belief that this intervention can cure patients with colorectal cancer, and that without it few survive 5 years. No good evidence exists supporting such convictions, indeed recent trial results challenge them. What evidence underpins this acceptance of illusory truths or misconceptions?

## Main

In this era of Covid-19 we have all been told to beware of fake and misleading news. Faulty information spreads as rapidly as the coronavirus itself and it is no accident that social media ‘likes’, YouTube ‘hits’ and Twitter ‘retweets’ are often described as going viral. Psychological studies have shown that opinions often outweigh facts.^1^ Constant repetition of the fake news, or any other process which increase the familiarity of falsehoods, either by design or the inadvertent misinterpretation of data, end up enhancing beliefs in the perceived truth of the misinformation.^2^ Individuals may draw inferences from consistencies with existing knowledge and source information stored in memory and are resistant to updating beliefs when facts change.

This phenomenon of illusory truth has been circulating in the world of oncology for years, namely about the benefits of pulmonary metastasectomy. The story is this: When a patient with colorectal cancer (CRC) has a few lung metastases their life will be prolonged by removing or ablating them and they might even be cured. And if they don’t have treatment their chance of surviving 5 years is 5% or less. This story is so widely believed that metastasectomy is becoming standard practice around the world and radiation oncologists and interventional radiologists are getting in on the act with stereotactic radiotherapy (SABR) and image guided thermal ablation (IGTA). Even the National Institute for Health and Care Excellence (NICE) in its latest update of the clinical guideline on CRC supports the practice.^3,4^ The condition of ‘oligometastatic disease’ (OMD) is widely discussed but has no agreed definition. It is a condition defined by therapeutic opportunity rather than any evidence from tumour biology.^5^

The ‘evidence’ supporting pulmonary metastasectomy for CRC patients has come from many published retrospective case series included in a meta-analysis of the 24 largest studies including 2589 patients with a 5-year survival of these patients of 42%.^6^ But this and all the other series lacked any comparable control population and are subject to both selection bias and immortal time bias. The widespread belief is that without metastasectomy these patients have a probability of survival of less than 5%—a belief echoed in a very recent letter to the BMJ.^7^

Only one randomised trial has tried to directly address this question—PulMiCC.^8^ Unfortunately, it had to close early because of slow recruitment (reflecting a general lack of equipoise on the question), and did not have sufficient power to demonstrate a 10% improvement in 5-year survival. It showed no significant difference in overall survival (HR 0.82, 95% CI 0.43, 1.56) but importantly the 5-year survival of the control group was 29% (95% CI 16–52%).^8^ There are two other randomised Phase 2 trials that have investigated ablation of metastases with overall survival as the primary outcome. The CLOCC trial^9^ randomised patients with liver metastases from CRC to IGTA or not and SABR-COMET^10^ randomised patients with metastases at a variety of sites (47% pulmonary) from a variety of tumours to have SABR or not. Both trials were small (119 and 99 patients, respectively) but appeared to show a benefit from intervention. The hazard ratio in CLOCC was 0.58 (95% CI 0.38–0.88) and in SABR-COMET 0.57 (95% CI 0.30–1.10). But despite randomisation, inappropriate stratification led to both trials showing imbalance in key prognostic factors favouring intervention, in particular in the proportions of participants with single rather than multiple metastases—the single most important prognostic factor. In SABR-COMET there was also a preponderance of patients with breast and prostate cancer, more likely to have a better prognosis. But even so the control patients had 5-year survivals of 25% in CLOCC and 29% in SABR-COMET.

So, the only randomised trials of metastasectomy all show that the survival of control patients is in the region of 25–29%—not the 5% or less—, which is so widely believed and quoted. These are not trivial and risk-free interventions. PulMiCC showed a significant decrease in lung function in those having surgery; CLOCC did not fully report adverse events but there seems to have been at least one IGTA-related death; SABR-COMET showed that 29% of SABR patients experienced Grade 2 or worse adverse events including three (4.5%) treatment-related deaths. All those involved in managing these patients need to acknowledge this and adjust their prior beliefs.

It is surely time for thoughtful clinicians to reflect on the shaky evidence that underpins this widespread and increasing practice and to rein in their enthusiastic colleagues. We need large definitive trials with overall survival as the primary outcome to determine whether there is in fact any benefit from removing or ablating metastases from any tumour type at any site, and if so, for which patients. Let us ignore the fake news or illusory truths and get some reliable evidence. Otherwise we may continue to waste resources, give false hope and cause needless harm.

## Data Availability

Not applicable.
